# Case Report: Tyrosine Kinase Inhibitors Induced Lymphadenopathy in Desmoid Tumor Patients

**DOI:** 10.3389/fendo.2022.794512

**Published:** 2022-03-23

**Authors:** Sotirios Papadopoulos, Pantelis Koulouris, Claire Royer-Chardon, Georgia Tsoumakidou, Ana Dolcan, Stephane Cherix, Maurice Matter, Patrick Omoumi, Antonia Digklia

**Affiliations:** ^1^ Department of Oncology, Centre Hospitalier Universitaire Vaudois, Lausanne, Switzerland; ^2^ Department of Pathology, Centre Hospitalier Universitaire Vaudois, Lausanne, Switzerland; ^3^ Department of Radiology, Centre Hospitalier Universitaire Vaudois, Lausanne, Switzerland; ^4^ Faculty of Biology and Medicine, University of Lausanne, Lausanne, Switzerland; ^5^ Department of Orthopaedics and Traumatology, Centre Hospitalier Universitaire Vaudois, Lausanne, Switzerland; ^6^ Department of Visceral Surgery, Centre Hospitalier Universitaire Vaudois, Lausanne, Switzerland

**Keywords:** case report, tyrosine kinase inhibitor, pazopanib, desmoid tumors, lymphadenopathy, side effects

## Abstract

Tyrosine kinase inhibitors (TKIs) are nowadays a valuable treatment of desmoid tumors, a rare mesenchymal neoplasm. Although many side effects of imatinib and pazopanib, commonly or rarely occurring, have been described, reactional lymphadenopathy has not yet been reported. In this publication, we report two cases of patients with desmoid tumors, treated with pazopanib and imatinib, who developed reactional lymphadenopathy. As this side effect is presented as a newly formed mass, it can result in new diagnostic questions and added imaging tests and can even lead to discontinuation of the treatment. This report may help the clinicians facing similar problems adopt a “watch and wait” approach.

## Highlights

- Multitargeted TKIs of vascular endothelial growth factor receptors are effective therapy for DT.
- We report on two patients with reactional adenopathy induced by imatinib or pazopanib, mimicking tumor progression or other primary tumors.

## Background

Desmoid tumors (DTs) are mesenchymal neoplasms considered to be of intermediate malignancy due to their non-metastasizing but locally invasive behavior (1–3). Their occurrence is rare, with an estimated incidence of 5 to 6 cases per million per year. Patients are typically young (peak age of 30–40 years), while there is a tendency toward females (1, 3). Its expected frequency of stabilization and reversal is 50%–60% and 20%–30%, respectively (2). Histologically, tumor cells are highly proliferating monoclonal mesenchymal stem cell progenitors arising in deep soft tissues (most commonly in the extremities of the thoracic wall, the abdomen, and the limbs) (3, 4), while the tumor’s surgical appearance is similar to that of tendons (Greek name “desmos”) (1). Clinically, DTs are characterized by great variability; symptoms depend on site, size, and progression speed and may include chronic pain, functional deficits, and ischemia, resulting in low quality of life and psychological burden (1, 2, 4). They may also be multifocal (1), and they often present continuous local reoccurrences, but they are generally not life-threatening (1, 3, 4). Their etiology is in most cases mutations of CTNNB1 gene, encoding beta-catenin pathway (sporadic form), while there is also a hereditary form associated with familial adenomatous polyposis (FAP) and APC gene mutations (1–4).

Tyrosine kinase inhibitors (TKIs) are orally administered anticancer drugs that inhibit tyrosine kinase proteins, a class of proteins that participate in various cellular processes (5). Pazopanib (Votrient^®^), a synthetic indazolylpyrimidine (6), is a small-molecule multitargeted TKI aimed at vascular endothelial growth factor receptor (VEGFR)-1, -2, -3, platelet endothelial growth factor receptor (PEGFR)-α, -β, fibroblast growth factor receptor (FGFR)-1, -2, mast/stem cell growth factor receptor (SCFR, c-KIT gene product), and partly colony-stimulating factor-1 receptor (CSF1R) (6–9). Common known side effects include, fatigue, vomiting, diarrhea, nausea hypertension, proteinuria, elevated alanine aminotransferase (ALT) and hepatic toxicity, neutropenia, leukopenia and anemia, left ventricular systolic dysfunction, hemorrhagic events, venous thromboembolic events, and gastrointestinal fistula, as well as other rare complications, which have been reported (9, 10). Imatinib (Gleevec^®^), a 2-phenylamino-pyrimidine, is also a multitargeted TKI, and it aims at PEGFR, SCFR (c-KIT gene product), and BCR-ABL fusion protein (5, 11). Common known side effects include abdominal pain/distention, ALT increase, alopecia, anemia, anorexia, arthralgia, aspartate aminotransferase increase, asthenia, blood creatinine increase, blood lactate dehydrogenase increase, bone pain, constipation, cough, depression, diarrhea, dizziness, dyspepsia, dyspnea, fatigue, flatulence, fluid retention, headache, hemoglobin decrease, hemorrhage [non-gastrointestinal/central nervous system (non-GI/CNS)], hypoalbuminemia, hypokalemia, hypoproteinemia infection, influenza, insomnia, joint pain, muscle cramps/spasms, musculoskeletal pain myalgia, nasopharyngitis, nausea, neutropenia, night sweats, pain, peripheral edema, periorbital edema, pharyngolaryngeal pain, pneumonia, pruritis, pyrexia, rhinitis, rigors, skin rash, sinusitis, thrombocytopenia, upper respiratory tract infection, vision, blurred, vomiting weight increase, and white blood cell decrease (11). As of June 2021, reactionary lymphadenopathy has not been reported as a side effect of these two TKI drugs in patients with DTs, while there is only one report about dasatinib, another TKI, in a patient treated in the context of chronic myeloid leukemia (CML) (12).

We are reporting the case of two patients with DTs treated with pazopanib in our department who presented reactionary lymphadenopathy after imatinib or pazopanib administration. The lymphadenopathy occurred as a new clinically palpable mass next to the known desmoid primary. MRI scans as well as biopsies were needed in order to exclude other possible diagnoses.

## Cases

Our first patient is female, aged 20 (born 2001), with no previous medical history. She was diagnosed with a right retroscapular DT located between the clavicle and the scapula with a p.T41A mutation of CTNNB1 gene (beta-catenin). She underwent surgical resection on November 2, 2011, and on December 6, 2012, a local recurrence was confirmed by biopsy. Due to rapidly symptomatic progression, chemotherapy according to the POG 9650 protocol (vinblastine i.v. and methotrexate i.v.) was introduced with partial remission of the disease. Four years later, on January 3, 2018, a new tumor progression appeared associated with local pain, and treatment by imatinib was started without any clinical and radiological benefit after 3 months.

**Figure 1:**
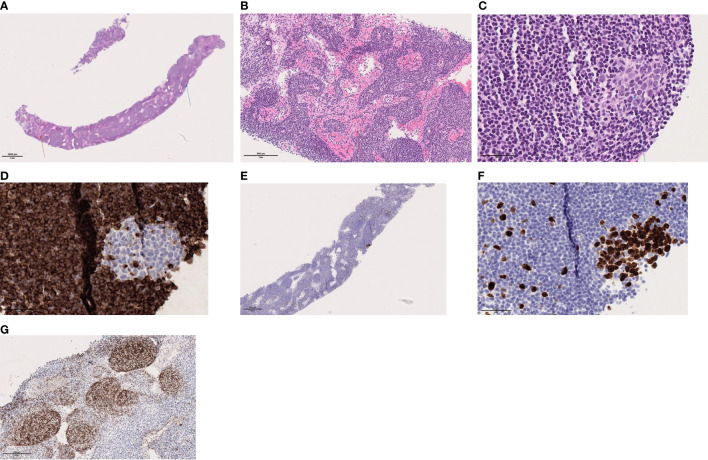
**(A)** Lymph node with preserved architecture, sinus histiocytosis (red arrow) and a germinal center (blue arrow), **(B)** Sinus histiocytosis, **(C)** Secondary follicle with germinal center, **(D)** BCL2 is negative within the secondary lymphoid follicle, **(E)** Ki64/MIB1: shows high proliferation in the germinal center within the secondary follicle; and low outside, **(F)** Ki64/MIB1: shows high proliferation in the germinal center within the secondary follicle and **(G)** CD1 shows the follicular dendritic network associated with the follicles. These are mainly primary follicles.

**Figure 2:**
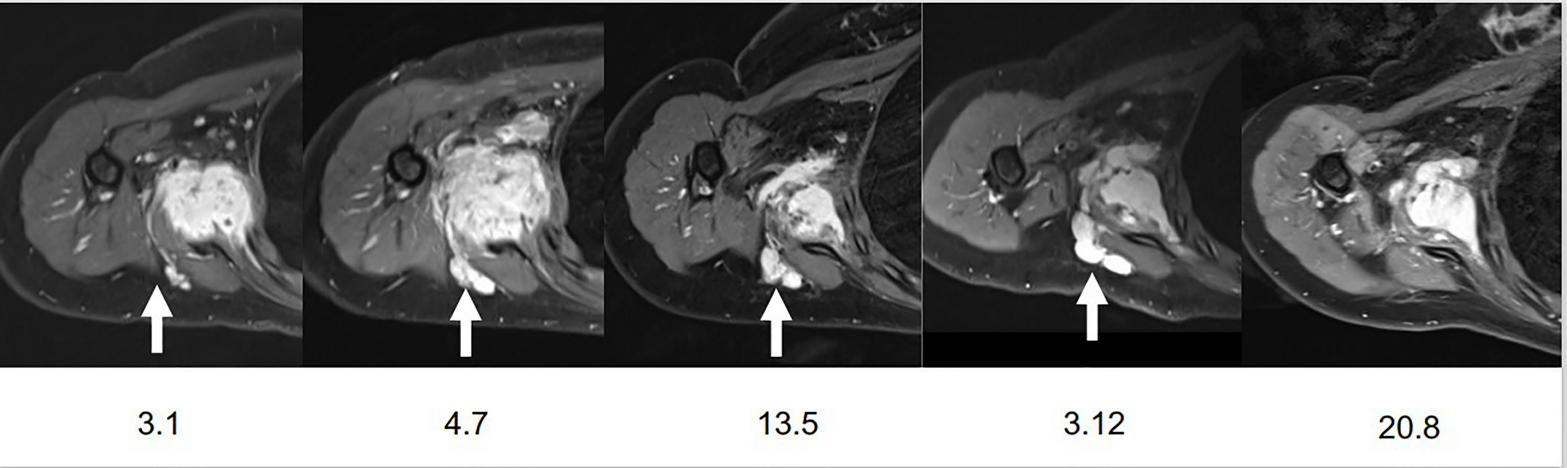
Evolution in time of the axillary lymph node.

In this context, treatment was switched for pazopanib on August 12, 2018, at an initial dose of 800 mg qd but a dose reduction to 600 mg qd due to abdominal pain. An MRI in November 2019 revealed the appearance of a nodular lesion, located between the teres minor and infraspinatus muscles, misdiagnosed as a new DT lesion. In March 2020, pazopanib treatment was stopped due to persistent abdominal pain. A follow-up MRI of the shoulder on December 3, 2020, revealed a tendency for progression of previous smaller lymph nodes, especially of the right lower axillary lymph nodes, compared to the two previous examinations as well as an increase of the nodular lesion. At that time and because of the dubious nature of this nodular lesion, we decided to do further investigations to rule out a possible disease progression. On January 14, 2021, the patient underwent a percutaneous biopsy and cryoablation of the right retroscapular polylobed target lesion with the question of a possible disease progression. Pathological examination of the retroscapular mass showed fragments of a lymph node of preserved architecture and with slight sinus histiocytosis; no sign of malignancy within sampling limits was found, proving thus that it was only reactionary lymphadenopathy (**Figure 1**). An MRI on March 8, 2021, by comparison to the examination on December 3, 2020, revealed an overall stability of the primary mass and a post-cryoablation status of the retroscapular lymphadenopathy (**Figure 2**).

Our second patient is female, aged 45 (born in 1976), known for obesity, obstructive sleep apnea syndrome, and moderate depressive episodes. She was diagnosed on November 4, 2020, with a sporadic rapidly increased right laterocervical DT of 4 × 10 × 5.8 cm with the presence of a c.121A>G (p.Thr41Ala) mutation in exon 3 of CTNNB1 gene.

**Figure 3:**
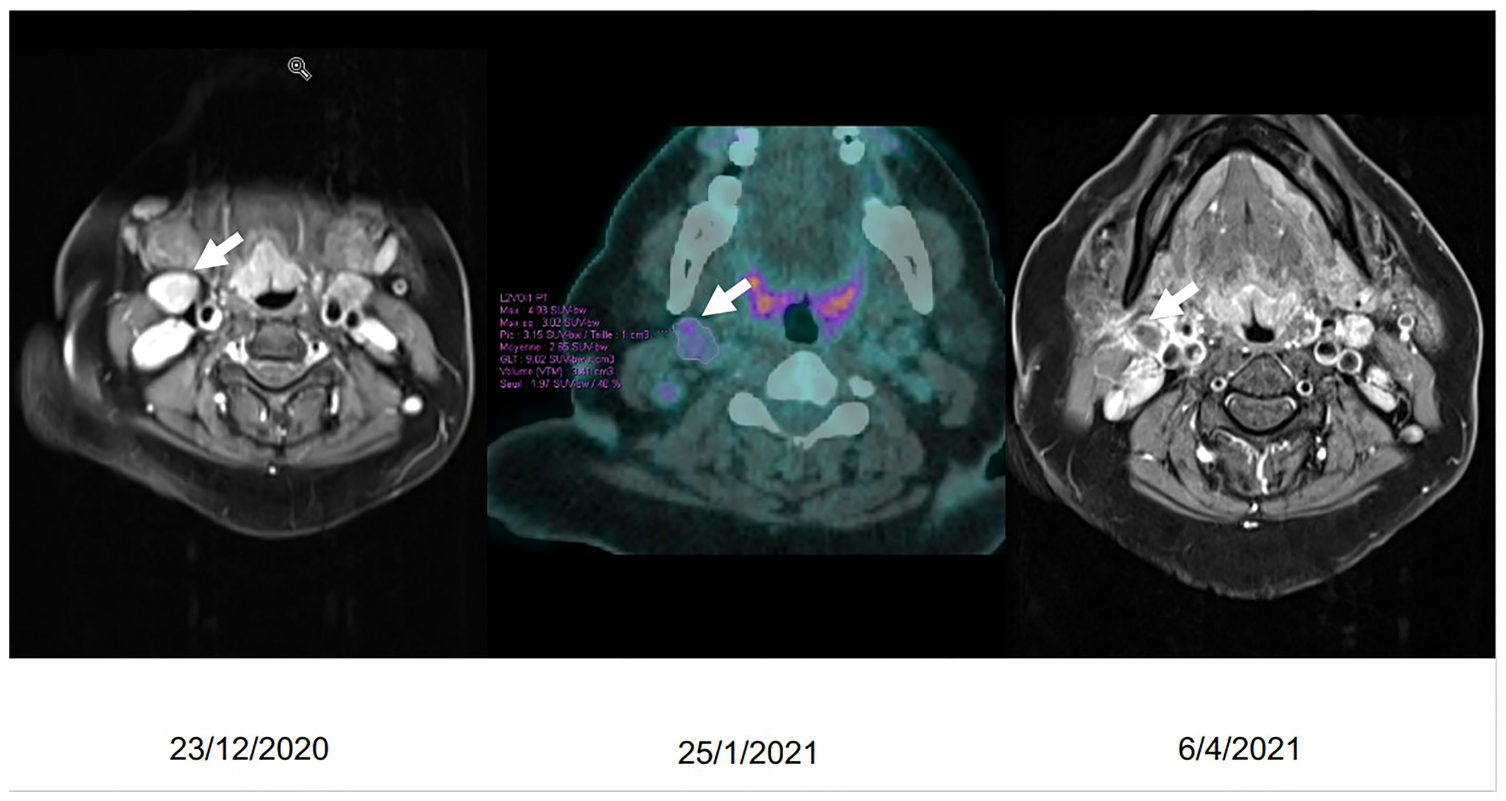
Evolution in time of the laterocervical lymph node.

Pazopanib treatment was introduced on December 4, 2020, at a daily dose of 800 mg qd. By the end of December 2020, she presented B symptoms (night sweats and fever); and at the clinical exam, we noted the appearance of palpable cervical lymph nodes on the right side. A PET-CT scan was demanded with the question of a possible second primary tumor (low-grade lymphoma?). It was performed on January 25, 2021, and it showed the known laterocervical mass as well as discrete hypermetabolism of peri-centimetric lymph node formations of the cervical area. Unable to exclude the presence of a low-grade lymphoma based on radiological imaging, we decided to perform a surgical biopsy. Our main motivation was the clinical image of the patient who was presenting persistent B symptoms. The surgical excision of a cervical lymph node (right cervical localization, level IIa/Ib) took place on February 22, 2021, and the pathological examination revealed benign lymphadenopathy associating follicular and paracortical lymphoid hyperplasia, sinus histiocytosis, moderate polytypic plasmacytosis, and hemorrhagic lysis in places. No metastatic tumor infiltration was found. A follow-up MRI on April 6, 2021, revealed a discrete decrease in the size of the primary tumor but a marked decrease in signal intensity and enhancement as well as a decrease in dimensions of the cervical lymph nodes (**Figure 3**). Our patient is still on pazopanib treatment to this day with good clinical tolerance and tumor stability on imaging.

## Discussion

DTs is a rare disease (1, 2, 13) associated with the highly variable clinical course (2, 4). Current consensus recommendations suggest a “watch and wait strategy” as an upfront approach, and medical treatment is proposed only on symptomatic progressive DTs (13). Surgery is not considered now to be the gold standard option because of the high rate of recurrences (4). Regarding systematic therapy, low-dose chemotherapy was for years an effective treatment leading to interesting response rates and long-lasting responses. In the last years, TKIs such as pazopanib and sorafenib have been found to present a combination of manageable toxicity profiles and good overall response rates (14). Nevertheless, the overall response rate is still comparable to that of chemotherapy (2, 13).

We have summarized above the most common side effects of pazopanib and imatinib, some of which can result in dose reduction or even discontinuation of the treatment. There are also reports for more rare complications, such as pancreatitis, posterior reversible encephalopathy, or periosteal reaction (10).

As far as we know, lymphadenopathy has not been reported as pazopanib- or imatinib-induced reaction. As mentioned above, reactional lymphadenopathy progressed in our two patients after the introduction of TKI treatment and was presented as a new nearby mass. This mass could at first be a second primary tumor, a progression of the known DT, a low-grade lymphoma (most difficult to be excluded without a surgical biopsy), and of course reactional lymphadenopathy since DTs do not spread to lymph nodes. This can perhaps lead to new diagnostic questions or even lead to treatment discontinuation.

In conclusion, TKIs are a valuable medication for DTs, and possible lymphadenopathy induced by the medication could lead to extra cost, operational dangers for the patient, and even treatment changes. We think that this two-case report can help clinicians facing similar problems to be less concerned with this kind of reaction and perhaps choose monitoring versus interventional acts. Nevertheless, more data are needed not only to validate the reactional lymphadenopathy as a side effect of TKI but also to determine the chance of its occurring and the possible relation to DT treatment.

## Data Availability Statement

The original contributions presented in the study are included in the article/supplementary material. Further inquiries can be directed to the corresponding author.

## Ethics Statement

The studies involving human participants were reviewed and approved by CHUV. The patients/participants provided their written informed consent to participate in this study.

## Author Contributions

ADi contributed to the conception and design of the study. SP wrote the first draft of the manuscript. PK wrote sections of the manuscript. All authors contributed to manuscript revision and read and approved the submitted version.

## Conflict of Interest

The authors declare that the research was conducted in the absence of any commercial or financial relationships that could be construed as a potential conflict of interest.

## Publisher’s Note

All claims expressed in this article are solely those of the authors and do not necessarily represent those of their affiliated organizations, or those of the publisher, the editors and the reviewers. Any product that may be evaluated in this article, or claim that may be made by its manufacturer, is not guaranteed or endorsed by the publisher.

## Abbreviations

DT, desmoid tumor; FAP, familial adenomatous polyposis.
